# Osteoprotective Effect of Enamel Matrix Derivatives on the Regeneration of Mandibular Defects in Experimentally Glucocorticoid-Induced Osteoporosis

**DOI:** 10.1155/2021/8659010

**Published:** 2021-10-13

**Authors:** Laila E. Amin, Naglaa Salama

**Affiliations:** ^1^Associate Professor of Oral Biology, Faculty of Dentistry, Mansoura University; Associate Professor of Oral Biology, Faculty of Dentistry, Horus University, Egypt; ^2^Associate Professor of Oral Pathology, Faculty of Dentistry, Mansoura University; Associate Professor of Oral Pathology, Faculty of Dentistry, Horus University, Egypt

## Abstract

**Purpose:**

Osteoporosis is a progressive systematic skeletal illness characterized by low bone mineral density and susceptibility to fracture caused by bone resorption. *Aim of the Study*. This study intended to evaluate the possible role of emdogain in combination with calcitonin on the healing of surgically induced mandibular defects performed on osteoporotic rats.

**Materials and Methods:**

Forty healthy female white albino rats were included in this study and divided into four groups. In group I (negative control), 10 rats received a vehicle injection after which a unilateral mandibular defect was created in each rat of all groups. Three groups were subjected to induction of osteoporosis by subcutaneous injection of 0.1 mg/kg/day dexamethasone for 60 days. In group II, rats were kept without treatment. In group III, rats were treated with daily intramuscular injection of 2.5 IU/kg of synthetic salmon calcitonin. In group IV, rats were handled as group III, and the created cavity was filled with emdogain. Rats were euthanized at 2nd and 4th week postsurgically. Hematoxylin and eosin, Masson's trichrome, NF-*κ*B (nuclear factor of activated B cells), and immunohistochemical stains were used, followed by statistical analysis.

**Results:**

Group I showed normal stages of bone defects healing. Group II revealed the formation of granulation tissue with dilated blood vessels, while groups III and IV showed enhanced bone healing and proper collagen fibers. The percentage area of newly formed collagen fibers was significantly higher in group IV at 2nd week (13.96 ± 0.020%) and 4th week (16.95 ± 0.024%) than in group II (8.75 ± 0.015% and 10.29 ± 0.015%, respectively) and group III (12.93 ± 0.015% and 14.61 ± 0.021%, respectively), but was lower than that in group I (15.75 ± 0.015% and 17.49 ± 0.015%, respectively).

**Conclusion:**

The local application of emdogain combined with systemically injected calcitonin improves bone healing in surgically induced bone defects in osteoporotic rats.

## 1. Introduction

Osteoporosis is a multifactorial skeletal disease characterized by disruption of the microarchitectural building of bone and reduction in bone mass that results in bone fragility and fast fracture with age, and described late in life, its origins can be traced back to childhood. Several reasons can play a role in the progress of osteoporosis, which include calcium levels in the diet throughout the bone growth stages, daily life, and hormones [1].

Glucocorticoids are important treatment strategies that have been described for long times, due to their potent anti-inflammatory and immunosuppressive properties. Bone growth, remodeling, and consistency are all harmed by glucocorticoids. Cortisone's action began with osteoblasts, which reduces reproduction and damages differentiation and growth, resulting in diminished bone construction [2]. Salmon calcitonin formed of a 32-amino acid peptide. It reduces the bone resorption via adhering and stimulating the calcitonin receptor on osteoclasts. However, the capability of salmon calcitonin to decrease breaks is debatable because of reports displaying an indistinct dose-response correlation [3].

Bone retains remodelling by continuous bone formation and bone resorption. Accurate organization of ossification by osteoblasts and bone resorption by osteoclasts donates to physiological bone preservation. Bone remodelling arises over three well-systematized phases. Briefly, osteoclasts are initially formed to resorb bone, then reversely, osteoblasts are subsequently activated, and finally, new bone is formed to make up for the bone loss. Disturbed bone remodelling cycles and imbalanced activity of osteoclasts and osteoblasts are often associated with pathological bone disease, arthritis, and osteolysis [4].

Periodontitis is a multifactorial inflammatory disease of infectious origin of periodontal tissues which, if not properly treated, can lead to the destruction of the tooth-supporting tissues and finally tooth loss. Moreover, some evidence has recently demonstrated a strict bilateral relationship between periodontitis and several systemic diseases such as cardiovascular disease, rheumatoid arthritis, metabolic syndrome, and diabetes [5, 6].

The rejuvenation of alveolar bone damage due to tooth extraction, periodontal disease, and/or trauma has been proven to be an important task. A wide range of supplies and procedures have been established and assessed [7]. Improvement of bone development, enhancement of the properties of the formed bone, decrease of the time of procedures (mainly for implant treatment), making easy applied materials, and improvement of the efficiency of natural procedures are the principal aims of regenerative processes [8].

Emdogain is obtained from porcine embryonic tissues, and it contains a mixture of enamel matrix derivatives (EMDs). The major constituent of EMD is amelogenin, which is a family member of hydrophobic proteins derived from a single gene by alternative splicing and controlled postsecretory processing. Emdogain is an osteopromotive agent that stimulates the bone growth and renewal treatments [9]. Emdogain has been revealed to improve the osteogenic properties of bone marrow by raising the whole count of bone cells, improving the production of osteoblasts, stimulating cell differentiation, and motivating immigration and capability of osteoblasts, which can result in enhanced bone restoration [8].

In periodontal diseases, infiltrated leukocytes produce inflammatory mediators that affect the expression of both receptor activator of nuclear factor (NF)-kappa B ligand (RANKL) and osteoprotegerin (OPG) in osteoblasts and periodontal ligament. Changes in the relative RANKL/OPG expression ratio are considered indicative of cell or tissue capacity to regulate bone resorption in different pathological conditions, including periodontitis [10]. RANKL plays a key role in inducing bone resorption by osteoclasts. Noteworthy, the role of RANKL combined with OPG appears relevant in periodontal tissues for the regulation of bone remodeling during orthodontic tooth movement as well as root resorption. On the other hand, it is known that nuclear factor-kappa B (NF-*κ*B) plays a central role during inflammation by regulating RANKL expression in different cell types [6].

NF-*κ*B (nuclear factor of activated B cells) is a member of transcription factors, which were initially recognized as controllers of B-cell differentiation by their capacity to fix to the *κ*B place of the kappa light chain gene in B cells. NF-*κ*B exposure sequentially triggers the inherent and adaptive immune system in reaction to antigen and autoimmune stimuli, additionally to several features of common cellular functions [11]. The NF-*κ*B stimulation is decontrolled in another situation, comprising cancer, diabetes, and atherosclerosis, highlighting the comprehensive variety of actions it does in normal and disease cases [12]. The atypical activation of NF-*κ*B makes an involvement to emerging numerous autoimmune, inflammatory, and malignant diseases, for example, atherosclerosis, rheumatoid arthritis, multiple sclerosis, malignant tumors, and inflammatory bowel diseases [13].

This study was conducted to test and compare whether local and systemic delivery of both emdogain and calcitonin could ameliorate the detrimental effects of glucocorticoid-induced osteoporosis in surgically induced mandibular bone defects in rats, or not. The null hypothesis assumed that there was no difference between the two treatment modalities.

## 2. Materials and Methods

### 2.1. Study Design

The study used forty female white albino rats with an average weight of 200–250 g. The ethical committee for animal care permitted the animal management and experimental procedures, which followed the methods outlined in the guiding principles for the use of laboratory animals approved by Ethical Committee of Faculty of Dentistry, Mansoura University, Egypt Number (code number: 0206129) and complied to ARRIVE guidelines of reporting animal research. All rats were held in the same environments and had free access to food and water. The rats were surgically prepared for a mandibular bone defect, and then the animals were divided into four groups (*n* = 10):

  Group I (control group): rats were injected with saline intramuscularly once a week for 60 days before a unilateral mandibular bone defect; this group of rats had surgery with a bone defect that was left to recover naturally without any treatment
  Group II (diseased control): rats were subjected to induction of osteoporosis by subcutaneous injection of 0.1 mg/kg/day dexamethasone for 60 days (Epidron®4 mg/ml) and then unilateral surgical mandibular bone defects were made
  Group III: animals were held as those of group II, but the rats were injected daily with 2.5 IU/kg intramuscular injectable synthetic salmon calcitonin (Miacalcic® 100 IU) [14]
  Group IV: similar to group III, but the bone defects were filled with enamel matrix derivatives (Emdogain®, Biora, AB, Malmo, Sweden) [15]

### 2.2. Osteoporosis Induction

Rats Were Weighted and Then Received Subcutaneous Injection of 0.1 mg/kg/day Dexamethasone for 60 Days (Epidron®4 mg/ml) [16].

#### 2.2.1. Osteoporosis Confirmation

Bone mineral density (BMD) of the lumber spine femur was measured before/after osteoporosis induction by the dual-energy X-ray absorptiometry (DEXA) scan technique using GE-Lunar DEXA (GE Healthcare, USA) with small animal software [17].

### 2.3. Surgical Procedures

Surgical procedures were performed at Surgical Unit of Mansoura Experimental Research Center, Mansoura, Egypt. An intraperitoneal injection of xylazine (25 mg/kg body weight) and ketamine (75 mg/kg body weight) was used to anaesthetize all animals. Following anaesthesia, all animals' submental regions were clean-shaven, and the skin cover of this area was sterile with a disinfected cotton pellet dampened with Betadine^TM^ povidone iodine 10%.

A sharp incision was made horizontally extraorally on the submental regions of mandible by a surgical blade No. 15 fixed on scalpel handle No. 3, and the incision mark traversed central area of the mandible. Skin at the buccal diastema region was reflected by a mucoperiosteal elevator to uncover the alveolar bone.

A round bone defect was created using a rose head bur no. 2 directly fixed in the contra angle hand piece using a low-speed micromotor. The defect was measured to confirm the uniform size of 2 mm width and depth. The bone cavity was flooded by normal saline, and both sides of the incision were approached and stitched using 3/0 silk mounted on a half circle needle 3/8″.

### 2.4. Postoperative Care

After surgery, the rats were monitored for at least 30 minutes in a cage covered with electrical heating pads to preserve body temperature. Then, they were housed in a clean sterile cage and fed soft diet and regular food. Pain killer 0.2 ml intramuscular (IM) and antibiotics 0.5 ml/200 gm (IM) were administrated by veterinaries for three days after surgery, and some animals took additional antibiotic dose for another two days, as determined by veterinaries.

The scarification time was at 2nd and 4th week postsurgery, and 5 rats from every set were injected by over dose of diethyl ether.

### 2.5. Histological Analysis

The mandibular samples were dissected, fixed in buffered formalin for 4 h, decalcified in ethylenediaminetetraacetic acid (EDTA) solution, and inserted in paraffin. Sequential sections were cut at a thickness of 4 *μ*m. The samples were managed for routine hematoxylin and eosin (HE) staining, Masson's trichrome staining (for illuminating collagen fibers and newly formed bone), and immunohistochemical identification of NF-*κ*B (nuclear factor-kappa) for recognition of every probable cellular alteration.

### 2.6. Computer-Assisted Digital Image Analysis

Slides images were analyzed on Intel® Core I3® based computer using Video Test Morphology® software (Russia) with a specific built-in routine for area measurement and stain quantification. Ten random fields were analyzed for each group (two sections from each sample). Images were recorded in relation to desired stain hue for both Masson's trichrome and immunohistochemical stain NF-Kb, to measure the percentage area of newly formed collagen and positive immune reaction, respectively, in relation to total area.

### 2.7. Statistical Examination

Data were studied with Statistical Package for Social Science software computer program version 23 (SPSS, Inc., Chicago, IL, USA). The Shapiro–Wilk test and Levene's test were used to test the normality of distribution and the homogeneity of variance, respectively. Data were presented as mean and standard deviation. One-way analysis of variance (ANOVA) and Tukey were intended for comparing more than two different groups. For any of the used tests, results were considered statistically significant if *P* value ≤0.05 (probability value).

## 3. Results

### 3.1. DEXA Results (Table 1)

The concentration of minerals at femur and lumbar in rats after induction of osteoporosis was significantly lower than before induction (*P*=0.015, *P*=0.019, *P* = 0.019).

### 3.2. Hematoxylin and Eosin Stain Results

**Figure 1:**
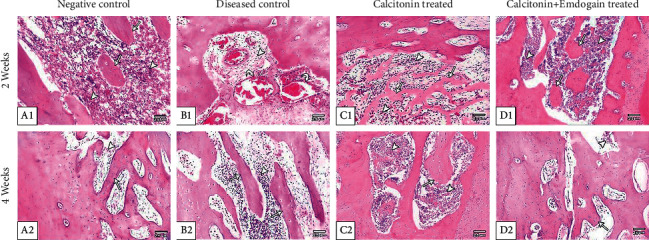
Photomicrograph showing (A1) the bone cavity filled with granulation tissue (arrow head) and the newly formed bone trabeculae (arrow) dispersed in rich highly cellular granulation tissue; (B1) the granulation tissue (arrow head) with severe accumulation of inflammatory cells and dilated blood vessels (curved arrows); (C1) the newly formed bone trabeculae (arrows) parallel to old bone and separated with highly cellular granulation tissue (arrow head); (D1) the newly formed bone trabeculae are anastomosing (arrows) and wide with highly cellular granulation tissue (arrow head); (A2) a net of growing new bony trabeculae (arrow) surrounded by moderately large bone marrow cavities (arrow head); (B2) the beginning of osteoid tissue formation (arrows) still surrounded by highly vascular granulation tissue (arrow head); (C2) the bone cavities were filled with regular bone trabeculae (arrow) formed of large size osteocytes enclosing bone marrow (arrow head); (D2) thick well-organized bone trabeculae (arrow) with osteoblasts lined the large bone marrow spaces (arrow head) (H&E staining ×100).

Two weeks after surgery: group I showed the cavity filled with woven bone, newly formed bone trabeculae dispersed in rich highly cellular granulation tissue, and definite reversal line demarcating the borders of the cavity. Group II revealed a large portion of cavity filled with granulation tissue with severe accumulation of inflammatory cells, dilated blood vessels, and small amount of bone spicules. Group III showed the woven bone filled with heavier bone trabeculae surrounded by large bone marrow cavities. Group IV showed anastomosing newly formed bone trabeculae surrounded by highly cellular large marrow cavities (Figure 1).

Four weeks after surgery: group 1 showed a net of growing new bony trabeculae surrounded by moderately large bone marrow cavities and defect was not completely filled. The trabeculae formed of wide, irregular size osteocytes. Group II showed increased quantity of formed bone trabeculae but of decreased connectivity and extended bone marrow cavities. Group III showed the bone cavities filled with regular bone trabeculae formed of large size osteocytes enclosing bone marrow. Group IV showed thick well-organized bone trabeculae with osteoblasts lined wide marrow spaces (Figure 1).

### 3.3. Histochemical Stain Results (Figure 2)

Masson's trichrome stains collagen fibers and a new bone matrix is stained blue; however, the well-calcified bone is stained red. Group I displayed reasonable blue stains at the 2nd week (15.75 ± 0.015); however, at the 4th week, the expression of blue color was increased (17.49 ± 0.0158) compared to group II at 2nd and 4th weeks (8.75 ± 0.015 and 10.29 ± 0.015, respectively). Group III sections showed adequate stain reaction at 2nd week (12.93 ± 0.0158). After 4 weeks, the bone tissue showed marked elevated calcification with properly arranged bone trabeculae (14.61 ± 0.024). In group IV, the expression improved at 2nd week (13.96 ± 0.024) and increased after 4 weeks (16.95 ± 0.24).

One-way ANOVA showed a significant difference among the study groups (*P* < 0.05). Post hoc analysis displayed significantly greater stain area percentage in the EMD-treated group compared to all other groups in all periods (Table 2).

### 3.4. Immunohistochemical Staining (Figure 3)

The positive immune expression of NF-*κ*B after 2 weeks showed a significant increase in mean ± SD results in group II (3.12 ± 0.021), group III (2.23 ± 0.011), and group IV (2.17 ± 0.027) in comparison with the negative control group (1.41 ± 0.019). Also the positive immune expression of NF-*κ*B after 4 weeks showed a significant increase in mean ± SD results in group II (2.71 ± 0.027), group III (1.42 ± 0.015), and group IV (1.39 ± 0.015) in comparison with the negative control group (1.25 ± 0.018).

The ANOVA test showed a significant difference throughout the study times. Furthermore, the post Hoc Tukey test at 2 weeks exhibited a significant difference (*P* < 0.0001) between groups I and II and group III conversely. Besides, there was a significant difference (*P*=0.002) between groups III and IV. After 4 weeks, the expression of NF-*κ*B showed a significant difference (*P* < 0.0001) between groups I and II and group III. Also, there was a nonsignificant difference (*P*=0.117) between groups III and IV (Table 3).

## 4. Discussion

Glucocorticoid-induced osteoporosis (GIO) is considered the main source for secondary adults' osteoporosis. GIO rat model was effectively recognized in the existing experiment after subcutaneous injection of dexamethasone for 60 days. The femur and lumber spine BMD of the rats after receiving the drug were significantly lower than BMD measurements of the same rats before receiving the drugs. These results are in line with previous studies which showed that prolonged treatment with glucocorticoid was related to low BMD and BMC with decreased regeneration and increased liability of bone fractures [18].

Bone repair is a highly compound mechanism that relies on the concerted intervention of many cell types and a series of vital proceedings, and it has long been regarded as the most important therapeutic goal [19]. NOD-like receptor (NLR) family detects molecular patterns in the cytosol and activates the formation of a multiprotein signaling platform, termed the inflammasome. The NLRP3 inflammasome, which is the best-characterized member of the inflammasome family, is a multiprotein complex that induces IL-1b and IL-18 inflammatory cytokine maturation by activating caspase-1 [20]. Periodontal diseases have been associated with increased bone resorption, and various studies support the relationship between inflammatory cytokines and RANKL-stimulated osteoclast activity. IL-1b and IL-18 are strong stimulators of *in vivo* and *in vitro* bone resorption via RANKL upregulation that stimulate osteoclastogenesis. Meanwhile, OPG inhibits osteoclast differentiation by binding to RANKL [4].

The present experiment assessed the efficiency of EMDs in combination with calcitonin in the healing of surgically induced bone cavity in the mandible in rats. The H&E histological results for the negative control group showed normal stages of bone defect healing. Group II showed obvious reduction in the quantity and quality of the regenerated bone tissue with the formation of granulation tissue, severe accumulation of inflammatory cells, dilated blood vessels, and small bony spicules, and these results are explained by the use of high doses of corticoid, which are associated with damaging effects on jawbone construction, repair, and consistency. Initially, cortisone's action began with osteoblasts, which reduces reproduction and damages differentiation and maturation, causing diminished bone turnover [19].

In the present study, group III treated with daily intramuscular injection of calcitonin showed faster healing process through newly formed bone spicules that were greater than that seen in the control positive group during each interval. CT is a 32-amino acid peptide hormone made mostly in the thyroid gland, and its receptor (CTR) is commonly identified for their capacity to control osteoclast-mediated bone resorption and increase calcium evacuation through the kidney. CT has also been revealed to inhibit osteoclast differentiation from originator cells and merging of mononucleated blood monocytes to form multinucleated blood monocytes [15].

The alteration in bone structure may be a main result of steroid therapy. Furthermore, increased osteolysis may be due to secondary hyperparathyroidism, which is produced by reduced intestinal calcium absorption and elevated urinary calcium excretion. As a result, CT is considered an effective controller of osteoclast-mediated bone resorption, making it a valuable treatment for osteoporosis [21].

The current work showed that group IV treated with EMDs displayed earlier repair of bone defects, which is in accordance with those of many authors who stated that EMD improves bone rejuvenation of rat femurs and also it accelerates new bone formation in rat skull defects. EMDs have been showed to have a significant effect in prompting the cell actions of several cells by assisting cellular interaction, proliferation, distribution, persistence, and differentiation, in addition to the activation of transcript factors, cytokines, and growth factors, which are included in controlling bone production [9].

Emdogain is a mixture of enamel matrix derivatives (EMDs) that can be used as an osteopromotive agent for bone augmentation/regeneration treatments. EMDs composed of a hydrophobic enamel matrix protein complex made from 6-month-old porcine tooth buds comprising about 90% amelogenin in addition to enamelin, tuftelin, tuft proteins, ameloblastin, and extra peptides such as bone morphogenetic proteins (BMPs) and transforming growth factor-beta (TGF-*β*) [8]. Tonetti et al. reported the role of topical application of EMD on the healing, surgical morbidity, and patient observation in profound intrabony defects. The outcomes were detected in early regeneration and tissue thickness after the use of EMD. Effective treatment of palatal radicular groove and the associated large intrabony cavity was completed with the administration of enamel matrix derivative without endodontic treatment or retreatment [22].

The histomophometric analysis percentage of collagen fibers in the negative control group showed regular repair of bone defect. The positive control group showed evident decrease in the amount of the restored bone tissue, and this was more recognized by the significantly reduced reaction to Masson's trichrome stains in comparison with other groups. According to Ignjatovic et al., osteoporosis is a condition characterized by alterations in bone structure and quantity that has become one of the most common health problems due to its widespread prevalence. As evidenced by the findings of numerous studies, system osteoporosis leads to complete destruction of the mandibular bones [23].

The current results of group III exhibited the significant action of calcitonin in increasing the amount and growth of the renewed bone. Other studies reported that the influence of calcitonin on the regeneration of the metaphysis bone could assist in the improvement of high-quality bone in the diaphysis, while cortical bone has a superior quality than that noticed in animals that were not treated with calcitonin [24]. Calcitonin gene-related peptide (CGRP), a neuropeptide that improves new blood vessel formation and callus growth, bone matrix formation, and calcification, along with BMSC recruitment and osteogenic differentiation, may be responsible for the enhanced bone formation mediated by calcitonin [25].

The properties of rejuvenated bone in the EMDS-treated group were better compared to other analyzed groups, including the CT group, based on histological data and statistical analysis. A derivative of the enamel matrix has been found to exhibit osteoinductive characteristics. The use of an enamel matrix derivative has been shown to increase the gene expression in human bone cells when it comes to the creation of extracellular matrix [26]. Previous research found that using an enamel matrix derivative only or a mixture of enamel matrix derivatives and deproteinized bovine bone mineral for the management of moderate bony defects had similar clinical and radiographic results after a year. Despite the restrictions imposed by the gel-like consistency, it has been hypothesised that enamel matrix derivatives can be utilised alone in periodontal regeneration, particularly in non-self-supporting defects [27]. Emdogain can stimulate osteoblasts and improve the formation of collagenase (i.e., matrix metalloproteinase-1), which damages matrix proteins in bone tissue microenvironments, leading to improved bone regeneration [8].

Immunohistochemical results in group II showed a significant increase in the immunoreaction of NF-*κ*B. Steroids at high doses promote the production of osteoclasts. Osteoblast signaling is disrupted, resulting in decreased osteoprotegerin release and enhanced nuclear factor-Kappa B ligand receptor activator, promoting the action of osteoclasts [28]. Furthermore, glucocorticoids form a destructive action on bone, decrease calcium absorption by the gastrointestinal tract, and increase renal calcium excretion. Steroids usually cause muscle weakness, which increases the risk of falls and fractures [29]. Boyce et al. stated that double knockout mice showed extreme osteoporosis due to the whole absence of osteoclasts. More research studies on these animals revealed that NF-B1 and NF-B2 are required for the differentiation of osteoclast precursors into osteoclasts relatively for persistence, which is a characteristic of NF-B activity [30].

Stimulation of NF-*κ*B arises in many cells via activation of a variety of stimuli-containing cytokines, immunological modulators, and further stressors. The important stimulants to NF-*κ*B comprise key activator of NF-*κ*B ligand (RANKL), TNF*α*, lymphotoxin, bacterial endotoxins, Toll-like receptor (TLR) ligands, CD 40L, interleukin-1 (IL-1), and oxygen radicals. Constantly, it was reported that the inhibition of NF-*κ*B was a significant method to prevent osteoclast development and bone resorption action. The discovery of the molecular machinery that activates NF-B allowed for the elimination of the pathway's main components in osteoclasts [31].

Calcitonin has been utilized in the treatment of osteoporosis and malignant humeral hypercalcemia. Many studies, on the other hand, show that calcitonin is involved in a variety of cellular activities, including cell survival, cell growth, and cell proliferation in a variety of cell types. It similarly plays a role in wound healing and affects cell invasion and metastasis in various cell lines. Calcitonin exerts its effects in part via regulating extracellular matrix (ECM) components, including type II collagen breakdown [32]. Fibronectin interacts with integrins to control numerous biological effects, including cell proliferation and death. Fibronectin expression is controlled by a number of transcription factors, comprising nuclear factor-kappa B (NF-B) and activator protein 1 (AP-1) [33].

The current study revealed a decreased expression of NF-*κ*B along the two examination periods. According to Miron et al., EMD was associated with a considerable reduction in interleukin-1 and a critical nuclear factor-kappa-B ligand receptor, as well as elevated expression of prostaglandin E2 and osteoprotegerin [34]. The receptor activator of NF-B ligand (RANKL) has been shown to be necessary for the stimulation of osteoclastogenesis. RANKL is formed by osteoblasts and bone marrow stromal cells, and its signal is transmitted by a particular receptor. Osteoprotegerin is a protein produced by osteoblasts and it inhibits osteoclastogenesis by competing with RANK and RANKL [35]. Through an elevation in OPG formation and a decline in RANKL release, EMD appears to play a potentially important role in the bone healing process. EMD can moreover stimulate the maintenance of newly formed mineral and the production of an encouraging osteogenic microenvironment that can particularly assist the growth of bone [36].

In terms of this study outcome, the null hypothesis is rejected in favor of EMD regenerative results that excelled at the histological level and other treatment modalities. However, EMD has a bright future, and further research and exploration are needed.

The lack of DEXA examination on mandibular bone and the lack of bone remodeling marker assessment are regarded as limitations of this study.

## 5. Conclusion

Our findings support the use of an exogenous factor such as EMD in the treatment of bone abnormalities when combined with salmon calcitonin in GIO. More research is needed to confirm our findings, which show that EMD could be effective as a bone promoter, and to develop a more biodisposable pharmaceutical form for intrabony usage.

## Figures and Tables

**Figure 2:**
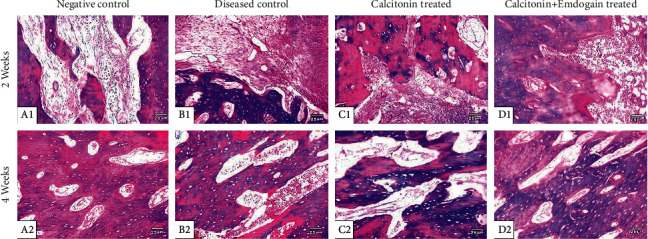
Histopathological sections of the 4 study groups at 2nd and 4th weeks; A1 and A2 represent the negative control group with newly formed collagen fibers radiating from the cavity periphery. B1 and B2 represent the positive control group which shows less Masson's trichrome reaction. More positively stained osteoid tissues in the calcitonin- and the calcitonin + emdogain-treated groups (C1 and D1 and C2 and D2, respectively) (trichrome stain, ×100).

**Figure 3:**
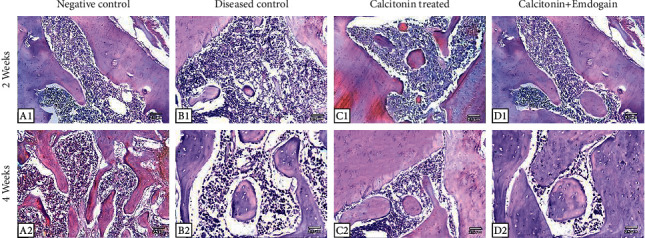
Immunohistochemical stain showed a reduced expression of NF-*β* in the negative control, calcitonin, and calcitonin + emdogain groups and increased expression of NF-*β* in the positive control group (×100, IHC).

**Table 1:** Comparison of femur and lumber spine BMD before and after osteoporosis using unpaired Student's *t*-tests.

		Before osteoporosis	After osteoporosis	*P*
Femur BMD	Mean ± SD	0.1407 ± 0.0287	0.1050 ± 0.008	0.015^*∗*^
Lumber BMD	Mean ± SD	0.1617 ± 0.01303	0.1203 ± 0.03406	0.019^*∗*^

SD: standard deviation; *P*: probability; ^*∗*^significant ˂0.05.

**Table 2:** Mean ± SD for the collagen fiber area percentage at two examination periods.

Percent area		Group I	Group II	Group III	Group IV	*P*
2nd week		15.75 ± 0.015	8.75 ± 0.015	12.93 ± 0.015	13.96 ± 0.020	<0.0001
	P1		<0.0001			
	P2			<0.0001		
	P3				<0.0001	

4th week		17.49 ± 0.015	10.29 ± 0.015	14.61 ± 0.021	16.95 ± 0.024	<0.0001
	P1		<0.0001			
	P2			<0.0001		
	P3				<0.0001	<0.0001

Data are expressed as mean ± SD for 5 replica each. SD: standard deviation; P: probability; test used: one-way ANOVA followed by the post hoc test for multiple comparisons; P1: significance relative to group I; P2: significance relative to group II; P3: significance relative to group III.

**Table 3:** Mean ± SD for the percentage area of NF-*κ*B at two examination periods.

Percent area		Group I	Group II	Group III	Group IV	*P*
2nd week		1.41 ± 0.019	3.12 ± 0.021	2.23 ± 0.011	2.17 ± 0.027	<0.0001
	P1		<0.0001			
	P2			<0.0001		
	P3				0.002	

4th week		1.25 ± 0.018	2.71 ± 0.027	1.42 ± 0.015	1.39 ± 0.015	<0.0001
	P1		<0.0001			
	P2			<0.0001		
	P3				0.117	<0.0001

Data are expressed as mean ± SD for 5 replica each. SD: standard deviation; P: probability; test used: one-way ANOVA followed by the post hoc test for multiple comparisons; P1: significance relative to group I; P2: significance relative to group II; P3: significance relative to group III.

## Data Availability

The SPSS data file used to support the findings of this study are available from the corresponding author upon request.

## References

[1] Lin X., Xiong D., Peng Y.-Q. (2015). Epidemiology and management of osteoporosis in the People’s Republic of China: current perspectives. *Clinical Interventions in Aging*.

[2] Ahmed R. Y., Elsherbini A. M., Elkhier M. T. A., Soussa E. F. (2020). A comparison of the early therapeutic effects of allogeneic bone marrow-derived mesenchymal stem cells and calcitonin on the healing of surgically induced mandibular bone defects in osteoporotic rats. *Archives of Oral Biology*.

[3] Turner A. G., Tjahyono F., Chiu W. S. M. (2011). The role of the calcitonin receptor in protecting against induced hypercalcemia is mediated via its actions in osteoclasts to inhibit bone resorption. *Bone*.

[4] Yamaguchi Y., Kurita-Ochiai T., Kobayashi R., Suzuki T., Ando T. (2017). Regulation of the NLRP3 inflammasome in porphyromonas gingivalis-accelerated periodontal disease. *Inflammation Research*.

[5] Isola G., Polizzi A., Santonocito S., Alibrandi A., Williams R. C. (2021). Periodontitis activates the NLRP3 inflammasome in serum and saliva. *Journal of Periodontology*.

[6] Currò M., Matarese G., Isola G. (2014). Differential expression of transglutaminase genes in patients with chronic periodontitis. *Oral Diseases*.

[7] Genco R. J., Williams R. C. (2010). *Periodontal Disease and Overall Health: A Clinician’s Guide*.

[8] Birang R., Abouei M. S., Razavi S. M., Zia P., Soolari A. (2012). The effect of an enamel matrix derivative (emdogain) combined with bone ceramic on bone formation in mandibular defects: a histomorphometric and immunohistochemical study in the canine. *The Scientific World Journal*.

[9] Izumikawa M., Hayashi K., Ali M., Polan A., Tang J., Saito T. (2012). Effects of amelogenin on proliferation, differentiation, and mineralization of rat bone marrow mesenchymal stem cells in vitro. *The Scientific World Journal*.

[10] Courtois G., Gilmore T. D. (2006). Mutations in the NF-*κ*B signaling pathway: implications for human disease. *Oncogene*.

[11] Vallabhapurapu S., Karin M. (2009). Regulation and function of NF- *κ*B transcription factors in the immune system. *Annual Review of Immunology*.

[12] Tilstra J. S., Clauson C. L., Niedernhofer L. J., Robbins P. D. (2011). NF-B in aging and disease. *Aging and Disease*.

[13] Mochizuki K., Inoue T. (2000). Effect of salmon calcitonin on experimental osteoporosis induced by ovariectomy and low-calcium diet in the rat. *Journal of Bone and Mineral Metabolism*.

[14] Cornelini R., Scarano A., Piattelli M. (2021). Effect of enamel matrix derivative (emdogain) on bone defects in rabbit tibias. *Journal of Oral Implantology*.

[15] Chen Z., Xue J., Shen T., Mu S., Fu Q. (2016). Curcumin alleviates glucocorticoid-induced osteoporosis through the regulation of the Wnt signaling pathway. *International Journal of Molecular Medicine*.

[16] Yuming L., Li Y., Yang W. (2007). Preventive effects of nitroglycerine on glucocorticoid-induced osteoporosis in growing rats. *Journal of Huazhong University of Science and Technology. Medical Sciences*.

[17] Samir S. M., Malek H. A. (2014). Effect of cannabinoid receptors 1 modulation on osteoporosis in a rat model of different ages. *Journal of Physiology and Pharmacology*.

[18] Isola G., Lo Giudice A., Polizzi A., Alibrandi A., Murabito P., Indelicato F. (2021). Identification of the different salivary Interleukin-6 profiles in patients with periodontitis: a cross-sectional study. *Archives of Oral Biology*.

[19] Ersozlu S., Sarisozen B., Ozer O., Adim S. B., Sahin O. (2017). The biochemical and histological analysis of subcutaneous calcitonin and intramedullary methylprednisolone on bone repair after bone marrow ablation: an experimental comparative study in rats. *Journal of Experimental Orthopaedics*.

[20] Pountos I., Georgouli T., Blokhuis T. J., Pape H. C., Giannoudis P. V. (2008). Pharmacological agents and impairment of fracture healing: what is the evidence?. *Injury*.

[21] Canalis E., Mazziotti G., Giustina A., Bilezikian J. P. (2007). Glucocorticoid-induced osteoporosis: pathophysiology and therapy. *Osteoporosis International*.

[22] Tonetti M. S., Fourmousis I., Suvan J., Cortellini P., Brägger U., Lang N. P. (2004). Healing, post-operative morbidity and patient perception of outcomes following regenerative therapy of deep intrabony defects. *Journal of Clinical Periodontology*.

[23] Ignjatovic N., Ajdukovic Z., Uskokovic D. (2005). New biocomposite [biphasic calcium phosphate/poly-DL-lactide-co-glycolide/biostimulative agent] filler for reconstruction of bone tissue changed by osteoporosis. *Journal of Materials Science: Materials in Medicine*.

[24] Lyritis G., Boscainos P. J. (2001). Calcitonin effects on cartilage and fracture healing. *Journal of Musculoskeletal & Neuronal Interactions*.

[25] Jia S., Zhang S., Wang X. (2019). Calcitonin gene related peptide enhances osteogenic differentiation and recruitment of bone marrow mesenchymal stem cells in rats. *Experimental and Therapeutic Medicine*.

[26] Weiss D. J., Bates J. H. T., Gilbert T. (2010). Stem cells and cell therapies in lung biology and diseases: conference report. *Annals of the American Thoracic Society*.

[27] Corbella S., Alberti A., Calciolari E., Taschieri S., Francetti L. (2019). Enamel matrix derivative for the treatment of partially contained intrabony defects: 12-month results. *Australian Dental Journal*.

[28] Sivagurunathan S., Muir M. M., Brennan T. C., Seale J. P., Mason R. S. (2005). Influence of glucocorticoids on human osteoclast generation and activity. *Journal of Bone and Mineral Research*.

[29] Suzuki S., Nagano T., Yamakoshi Y. (2005). Enamel matrix derivative gel stimulates signal transduction of BMP and TGF-{beta}. *Journal of Dental Research*.

[30] Boyce B. F., Xing L., Franzoso G., Siebenlist U. (1999). Required and nonessential functions of nuclear factor-kappa B in bone cells. *Bone*.

[31] Tsagaraki I., Phenekos C., Tsilibary E., Tzinia A. (2013). Calcitonin-induced NF-*κ*B activation up-regulates fibronectin expression in MG63 osteosarcoma cells. *Anticancer Research*.

[32] Ito S., Suzuki N., Kato S., Takahashi T., Takagi M. (2007). Glucocorticoids induce the differentiation of a mesenchymal progenitor cell line, ROB-C26 into adipocytes and osteoblasts, but fail to induce terminal osteoblast differentiation. *Bone*.

[33] Jia D., Yan M., Wang X. (2010). Development of a highly metastatic model that reveals a crucial role of fibronectin in lung cancer cell migration and invasion. *BMC Cancer*.

[34] Miron R. J., Dard M., Weinreb M. (2015). Enamel matrix derivative, inflammation and soft tissue wound healing. *Journal of Periodontal Research*.

[35] Mulholland B. S., Forwood M. R., Morrison N. A. (2019). Monocyte chemoattractant protein-1 (MCP-1/CCL2) drives activation of bone remodelling and skeletal metastasis. *Current Osteoporosis Reports*.

[36] Froum S. J., Weinberg M. A., Rosenberg E., Tarnow D. (2001). A comparative study utilizing open flap debridement with and without enamel matrix derivative in the treatment of periodontal intrabony defects: a 12-month re-entry study. *Journal of Periodontology*.

